# A Pilot Intervention for Antisocial Personality Traits in Male Prisoners: The Conditional Effect of Rule‐Breaking

**DOI:** 10.1002/brb3.71310

**Published:** 2026-03-12

**Authors:** Mustafa Özmen, Müjgan Kalabalık

**Affiliations:** ^1^ Vocational School of Health Services Bingöl University Bingöl Türkiye; ^2^ Bingöl Open Prison, Directorate General of Prisons and Detention Centres Ministry of Justice Bingöl Türkiye

**Keywords:** aggression, antisocial personality traits, experimental intervention, prisoners, rule‐breaking

## Abstract

**Background:**

Given that a significant proportion of the growing number of individuals in prisons may have antisocial personality traits (ASPT), it is important to test and report on the results of pilot psycho‐educational intervention programmes developed for this purpose.

**Aim:**

The primary aim of this study is to examine the effect of the psychoeducation programme on ASPT among prisoners. The secondary aim is to investigate whether there is any interaction between ASPT after intervention.

**Methods:**

Twenty‐six participants were randomly assigned to the experimental group (*n* = 13) and the control group (*n* = 13). The ASPT Scale was administered as a pre‐test and post‐test at 8‐week intervals. Findings were examined using independent samples *t*‐tests and repeated measures analysis of variance (ANOVA) via SPSS 27. PROCESS macro was used to examine whether reductions in the aggression subscale were moderated by rule‐breaking.

**Results:**

Group‐based psychoeducational intervention did not lead to effective results in overall ASPT scores in the experimental group. However, intervention led to significant decreases in aggression scores both within groups (*p* < 0.01) and between groups (*p* < 0.05). Furthermore, it was found that a decrease in aggression scores was moderated by rule‐breaking scores of the prisoners (*β* = 0.60, *p* < 0.05, [0.88; 1.12]).

**Conclusions:**

Group‐based psychoeducational interventions are associated with a reduction in aggressive behavior among prisoners. In experimental interventions aimed at reducing aggressive behavior, it is important to prioritize individuals with low levels of rule‐breaking tendencies.

## Graphical abstract

In this study, a psychoeducational intervention programme developed to reduce antisocial personality traits in male prisoners was implemented. The initial findings of the experimental intervention showed no significant reduction in the rule‐breaking, impulsivity, and lying sub‐dimensions, but did show a significant reduction in the aggression sub‐dimension. Furthermore, the significant reduction in aggression tendencies was significantly moderated by rule‐breaking.

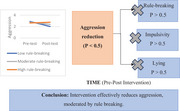

## 1 Introduction

According to the DSM‐5 (American Psychiatric Association (APA) 2013), antisocial personality disorder (ASPD) is characterized by difficulty adhering to legal or cultural normative values and a lack of empathy. The lifetime prevalence of ASPD in the general population ranges from 1% to 4% (Lenzenweger et al. 2007; Trull et al. 2010). Current studies indicate that ASPD rises to 3.91% in early adulthood and declines with age. It has also been reported that this disorder co‐occurs with many psychopathologies such as anxiety, depression, and addiction (Holzer et al. 2022; Moffitt et al. 2002). These results emphasize that other psychopathological processes should not be neglected in clinical interventions targeting antisocial personality organization. Furthermore, due to pathological levels of impulsivity and risk‐taking tendencies, individuals with antisocial tendencies are prone to committing crimes (American Psychiatric Association (APA) 2013). Individuals with ASPD are 2.44 times more likely to be charged with multiple types of offences (compared to individuals without this disorder) (Fridell et al. 2008). Therefore, it is thought that an intervention targeting ASPT could significantly reduce individuals' potential for involvement in crime. This study examined the effectiveness of an intervention targeting ASPT, which provides an important clue for ASPD.

ASPT can be categorized as aggressive (i.e., physical violence, fighting) and non‐aggressive behaviors (i.e., rule‐breaking, lying) (Bongers et al. 2004). A previous study reported that deceit plays a more central role among ASPT and has the potential to influence other personality traits (Machado et al. 2025). A network analysis study has strongly demonstrated that a lack of remorse or guilt perception plays a central role in ASPT (Gori et al. 2025). These results highlight the importance of studies based on the interaction between personality traits. This study reports the results of a pilot intervention for ASPT, with the aim of also reporting the potential interaction of personality traits. For example, research has revealed a clear relationship between aggressive impulses and rule violations (Bongers et al. 2004). Similarly, some behavioral theories report that ASPT, such as aggression and rule‐breaking, coexist in individuals (Jessor and Jessor 1977). All these findings indicate that the interaction between subcomponents, such as aggression and rule‐breaking, is noteworthy in psychological interventions targeting ASPT.

There is a growing body of literature on experimental interventions designed for ASPT (Brännström et al. 2016; van der Sluys et al. 2024). It has been found that Impulsive Lifestyle Counselling, developed as an alternative to traditional therapies, significantly reduces the overall crime rate among individuals with ASPD (Hesse et al. 2022). Although a psychoeducation programme designed for ASPT reduced substance dependence and aggression scores, it did not produce a significant effect (Thylstrup et al. 2015). Previous studies have shown that mentalization‐based therapies (MBT) yield effective results for ASPD and borderline personality disorder (BPD) (Bateman et al. 2016). Furthermore, other studies have reported that Dialectical Behavior Therapy produced effective results in men with ASPD and BPD (Wetterborg et al. 2020). Another study has shown that psychological interventions lead to meaningful results in both criminality and antisocial tendencies (Farrington et al. 2022). All these studies indicate that interventions targeting ASPT yield varying results. The fact that personality traits tend to stabilize with age (Cobb‐Clark and Schurer 2012) may limit the effectiveness of the intervention. However, meta‐analyses highlight that personality also has small but changeable traits (Bleidorn et al. 2022). In clinical studies, it is common for numerous factors to influence the process, such as the practitioner's professionalism, participants' resistance or openness to benefit from the intervention, the setting in which the intervention is conducted, and the content of the intervention itself. This study was conducted with a specific group possessing relatively unique characteristics (convicts). The findings obtained can therefore be interpreted as being limited to this group.

A recent study has reported that very few interventions have been developed and evaluated for prisoners (Andrade et al. 2024). The number of people in prisons in Türkiye reached approximately 380,000 as of 2024 (Adli Sicil ve İstatistik Genel Müdürlüğü 2024). It has been reported that approximately 96% of prisoners in Türkiye are male and that a significant proportion of crimes committed, 15.7% of which are assaults, can be linked to ASPT (Türkiye İstatistik Kurumu 2020). The aim of this study is to test the effectiveness of psychoeducation‐based group intervention for prisoners. Because individuals with ASPT are significantly more likely to be prosecuted for criminal offences (Fridell et al. 2008). Furthermore, as being male is a significant risk factor for ASPD (Schulte Holthausen and Habel 2018), the study was conducted in a correctional facility housing only males. An effective psychological intervention targeting ASPT can facilitate the integration of prisoners into society and prevent recidivism. Considering the above theoretical and empirical findings, the following hypotheses were tested: first, we examined the effectiveness of the intervention; that is, we assumed that psychoeducation would lead to effective reductions in all components of ASPT. Second, we investigated whether other personality traits had a moderating effect on any reduction in antisocial personality components.

## 2 Method

### 2.1 Procedure and Participants

Prior to commencing experimental interventions with prisoners in prison institutions, approval was obtained from the Bingöl University Social and Human Sciences Ethics Committee (Date: January 17, 2025; ID: 33117789/044/194244). Subsequently, two separate institutional permissions were obtained from the General Directorate of Prisons and Detention Centres, affiliated with the Ministry of Justice, at different times for the conduct and publication of the study. The inclusion criteria for the study were determined as follows: (a) having been convicted of a crime defined as a simple assault; (b) not having a psychotic diagnosis to a degree that would impair sensory integrity. As stated in the institutional permission, personal information such as names was not collected from the participants. Instead of participant names, pseudonyms or numbers were used. The data has been stored in a specially encrypted environment accessible only to the research team. After the research is published, the data will be destroyed and will no longer be accessible. Participants were informed that the study was voluntary and that they could withdraw from the study at any stage. No reward, money, or points were provided to participants as a result of their participation in the study. All stages of the research were conducted in accordance with the criteria set out in the Helsinki Declaration.

Thirty‐five male inmates at Bingöl Open Prison were enrolled in the study. The number of participants in the experimental intervention was calculated using G*Power 3.1.9.7 (Faul et al. 2007). In the study comparing independent groups, it was reported that the study could be conducted with a total of 26 participants, 13 in each group, based on the parameters of effect size *d* = 0.90 (large effect), significance level *α* = 0.05, and statistical power (1–*β*) = 0.70. Previous studies indicate that a sample group of 26 individuals is sufficient, as calculated using G*Power 3.1.9.7 (Balconi et al. 2025). A total of nine participants withdrew from the study following the establishment of the research protocol (*n* = 6) and the commencement of experimental interventions (*n* = 3). Participants were randomly assigned to experimental and control groups using computer software. The final sample consisted of 26 individuals, comprising an experimental group (*n* = 13) and a control group (*n* = 13). Participants' ages ranged from 21 to 62 years (*M* = 38.58). The length of time participants spent in prison ranged from 2 to 10 years (*M* = 4.22). The distribution of participants' criminal histories consists of the manufacture and sale of narcotics or stimulants (*n* = 4), theft (*n* = 6), and violent acts (*n* = 16).

## 3 Measures

### 3.1 Demographics

Participants provided their age and gender information

### 3.2 Antisocial Personality Characteristics Scale (APCS)

APCS (Bilge and Mayda 2023) is a 17‐item Likert‐type scale developed to assess an individual's antisocial personality tendencies. The scale is scored from 1 (*completely untrue for me*) to 5 (*describes me perfectly*). High scores indicate high antisocial personality traits. The APCS consists of four subscales: rule‐breaking, impulsivity, aggression, and lying. Sample item: “Nothing can stop me from doing what I want.” The reliability coefficients of the scale's subscales range from 0.67 to 0.76, which is above the acceptance threshold. Furthermore, it has been reported that the correlation in the test‐retest reliability results, conducted at 3‐week intervals, ranged between 0.72 and 0.89. There is no cut‐off point in APCS that could provide clinical inference.

### 3.3 Beck Hopelessness Scale (BHS)

BHS (Beck et al. 1974) was developed to assess individuals' negative expectations about themselves and the future, and adapted into Turkish (Durak and Palabıyıkoğlu 1994). The BHS consists of 20 items and is scored from 1 (yes) to 2 (no). Example item: “The future seems unclear and uncertain to me.” Raw scores obtained from this structure range from 0 to 5. As scores increase, the level of hopelessness increases. Previous studies have shown that Cronbach's alpha (*α* = 0.84) is good for the reliability of the scale (Özmen 2025).

### 3.4 Intervention

Informed consent forms prepared in writing by the participants were signed, and participant consent was formally obtained. Participants were first informed about the purpose, scope, and possible outcomes of the intervention. It was stated that during the 8‐week implementation period, they could withdraw from the study at any stage without having to provide any reason or excuse. It was emphasized that the entire psychoeducation process was planned as group work. Subsequently, measurement tools were applied for the pre‐test. Following the pre‐test interventions, an 8‐week programme was conducted with participants randomly selected for the experimental group. A special online platform was used for random assignments. The sessions conducted during the programme were structured as psycho‐educational group work. The structured pilot intervention was conducted by the second author, who works as a psychologist in correctional facilities. The second author is trained in anger management, empathy skills acquisition, and impulsive behavior training aimed at improving antisocial personality tendencies. The members of the experimental group were ensured to participate in all stages of the intervention. No intervention was performed on the control group.

The content of the 8‐week pilot psycho‐educational programme for prisoners is structured as follows. The first 2 weeks of the intervention training covered the definition and characteristics of ASPT, as well as its biological, psychological, and social features. The 3rd and 4th weeks discussed the thought processes of individuals with ASPT and their effects on emotions and behaviors. Exercises aimed at understanding the feelings and thoughts of others (i.e., empty chair) were also conducted. In the 5th week, after defining impulsive behaviors such as aggression and rule‐breaking, role‐playing was used to try to develop alternative behavior skills by teaching control skills. The 6th week addressed the societal consequences of crime and rule violations. The functionality of society's normative values was examined, and possible real‐life scenarios related to violence and rule‐breaking were discussed. The 7th week presented practical examples of effective coping methods for problems, as well as effective and healthy communication strategies. The final session was structured to encourage active participation from attendees; it was supported by question‐and‐answer activities, discussions of examples from daily life, and guidance on how the information could be integrated into life after prison. Furthermore, connections were made between the topics covered in psychoeducation to facilitate integration into life after prison and daily life.

### 3.5 Analysis Strategy

Evaluator blinding was applied to counteract potential biases that could affect the results. All analyses in the study were conducted using SPSS 27. Independent samples *t*‐tests were applied to examine whether there was any age‐related variation before assigning participants to groups. To understand whether the data met the assumptions for parametric analyses, skewness (> ±2) and kurtosis (> ±7) non‐normal criteria were taken into account (Kim 2013). Subsequently, the pre‐test and post‐test score changes in hopelessness levels, one of the strongest indicators of depression among factors that could be effective in interventions targeting ASPT, were examined. Findings regarding intergroup differentiation and time‐related change (pre‐test and post‐test) were examined using repeated measures analysis of variance (ANOVA). Moderation analyses were tested using the PROCESS macro (version 3.5) model 1 (Hayes 2013). The assumption that the effects are significant was used if they did not include zero in the 95% confidence interval with 5000 resamples (Preacher and Hayes 2008).

## 4 Results

### 4.1 Preliminary Analyses

Before examining the differences in findings from the experimental and control groups, it was determined whether the data met the assumptions of normality. It was reported that skewness ranged from −0.46 to 1.41, kurtosis ranged from −1.44 to 2.32, and the assumptions of normality were met. Subsequently, a paired sample *t*‐test before and after the intervention revealed that participants' levels of hopelessness did not change significantly in either the experimental (*p* = 0.173) or control group (*p* = 0.323). In the pre‐test, all participants who took part did not show any age‐related differences in terms of ASPT according to the one‐way ANOVA test: rule‐breaking (F(1, 24) = 0.441, *p* = 0.919), impulsivity (F(1, 24) = 0.807, *p* = 0.670), aggression (F(1, 24) = 0.689, *p* = 0.753), and lying (F(1, 24) = 0.827, *p* = 0.656). Therefore, age was not a determining variable in assigning participants to the experimental and control groups. The findings for the experimental and control groups are reported in Table 1 using an independent samples *t*‐test.

**TABLE 1:** Descriptive characteristics of pre‐test and post‐test according to the intervention (experimental‐control) conditions.

Experimental (*n* = 13)				Control (*n* = 13)					
	Pre‐test	Post‐test	T0 T1	Pre‐test	Post‐test	T0 T1	Grup × Time		
	M (SD)	M (SD)	Pre‐post	M (SD)	M (SD)	Pre‐post	*F*	*p*	*η* ^2^
ASPT‐total	2.66 (0.30)	2.46 (0.72)	0.24	2.13 (0.87)	2.16 (0.74)	0.77	1.50	0.23	0.06
Rule‐breaking	2.28 (0.84)	2.09 (0.86)	0.50	1.90 (1.08)	1.95 (0.98)	0.76	0.56	0.46	0.02
Impulsiveness	3.00 (0.69)	2.81(1.09)	0.38	2.38 (0.92)	2.31 (0.80)	0.97	0.56	0.46	0.02
Aggression	2.75 (0.47)	2.35 (0.65)	0.01^**^	2.06 (92)	2.05 (0.85)	0.98	6.06	**0.02** ^*^	0.20
Lying	2.38 (0.88)	2.48 (0.95)	0.47	2.18 (0.74)	2.28 (0.86)	0.76	0.07	0.78	0.01

Abbreviation: ASPT, antisocial personality traits.

^*^ *p* < 0.05; ^**^ *p* < 0.01

### 4.2 Effectiveness of Intervention on ASPT

**FIGURE 1:**
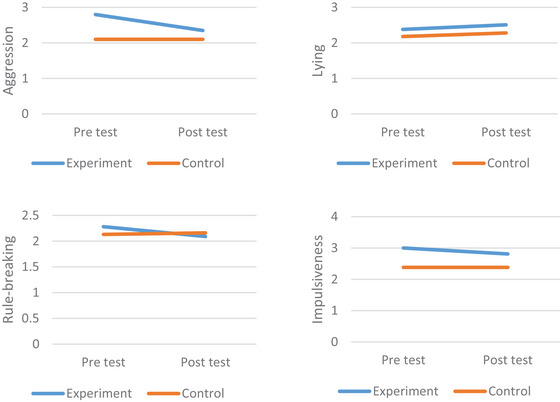
ASPT sub‐dimension score over time and by condition.

To examine whether the experimental intervention aimed at reducing ASPT produced a significant effect compared to the control group, Repeated measures ANOVA was used (see Table 1). The main effect of time (F(1, 24) = 1.54, *p* > 0.05) on the total antisocial score, as well as the time x intervention effect (F(1, 24) = 1.50, *p* > 0.05, *η* ^2^ = 0.06), were not significant. In the rule‐breaking subscale, the main effect of time (F(1, 24) = 0.48, *p* > 0.05) and the time x intervention interaction effect (F(1, 24) = 0.56, *p* > 0.05, *η* ^2^ = 0.02) were found to be non‐significant. In the impulsivity sub‐dimension, the main effect of time (F(1, 24) = 0.38, *p* > 0.05) and the time x intervention interaction effect (F(1, 24) = 0.56, *p* > 0.05, *η* ^2^ = 0.02) were found to be insignificant. In the aggression sub‐dimension, the main effect of time (F(1, 24) = 8.29, *p* < 0.05) and the time x intervention interaction effect (F(1, 24) = 6.06, *p* < 0.05, *η* ^2^ = 0.20) were found to be significant (see Figure 1). In the lying sub‐dimension, the main effect of time (F(1, 24) = 0.54, *p* > 0.05) and the time × intervention interaction effect (F(1, 24) = 0.07, *p* > 0.05, *η* ^2^ = 0.01) were found to be insignificant. Finally, the significant decreases in aggression scores may also be attributable to regression to the mean. This is because, following randomization, the pre‐test aggression scores for the experimental group were much higher than those for the control group. Therefore, the pre‐test scores were included in the model as a covariate, and pairwise comparisons analyses were repeated, confirming that the results remained significant (SE = 0.145, *p* < 0.05).

### 4.3 The Conditional Effects on Aggression

**FIGURE 2:**
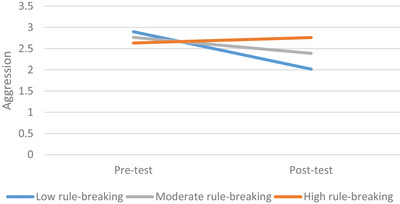
The interaction effect of rule‐breaking and time on aggression.

The PROCESS macro model 1 was used to examine whether the moderating effect of rule‐breaking exists in the effect of experimental intervention on aggression (Hayes 2013). After the pre‐test was checked, it was found that the significant effect of the experimental intervention on aggression was moderated by the individual's perception of rule‐breaking. The overall model (F(4, 21) = 3.78, *p* < 0.05, *R* ^2^ = 0.34) and the interaction effect (*β* = 0.60, *p* < 0.05, [0.88; 1.12]) were found to be significant (see Table 2). These results can be evaluated in several stages. First, the intervention significantly reduced aggression tendencies among prisoners. However, the decrease in aggression tendencies occurred strongly among prisoners with low perceptions of rule‐breaking (*β* = −0.88, SE = 0.29, 95% CI = [−1.48, −0.27]), while it occurred weakly among prisoners with medium perceptions of rule‐breaking(*β* = −0.38, SE = 0.20, 95% CI = [−0.80, 0.04]) and high (*β* = 0.13, SE = 0.29, 95% CI = [−0.47, 0.73]) levels of perceived rule‐breaking. Figure 2 shows the graphs of the slopes for individuals with low, medium, and high levels of rule‐breaking tendencies.

**TABLE 2:** Slopes of time (pre‐test vs. post‐test) predicting aggression at each level of rule‐breaking.

Different RB values	*β*	SE	*t*	95% CI
−0.84 (−1.84)	−0.88	0.29	−3.02	[−1.48; −0.27]
0.00 (M)	−0.38	0.20	−1.85	[−0.80; 0.04]
0.84 (1.84)	0.13	0.29	0.44	[−0.47; 0.73]

Abbreviations: CI, confidence Interval; RB, rule‐breaking.

## 5 Discussion

The present study investigated the effectiveness of psychoeducation‐based group interventions developed for prisoners in prison. Prisoners were randomly assigned to either a 4‐week experimental group or a control group. Consistent with our first hypothesis, it demonstrated a reduction in the total effect of ASPT and in each subcomponent (except for the lying subcomponent). However, group‐based psychoeducation was found to have a strong, significant effect only on the aggression sub‐dimension. Therefore, the first hypothesis was partially confirmed. Consistent with our second hypothesis, the decrease in the aggression sub‐dimension was moderated by the rule‐breaking sub‐dimension. Therefore, our second hypothesis was confirmed. In fact, both hypotheses were tested in a related manner. The results of the second hypothesis were shaped based on the results obtained in the first hypothesis. In other words, the significant decrease in aggression tendencies in the first hypothesis was moderated by the interactive effect of rule‐breaking in the second hypothesis, with the effectiveness of psychoeducation.

ASPT significantly increase the risk of individuals becoming involved in criminal offences (Fridell et al. 2008). This risk differs significantly in a positive direction in men (Schulte Holthausen and Habel 2018). Furthermore, the dramatic increase in the number of prisoners in Türkiye (Adli Sicil ve İstatistik Genel Müdürlüğü 2024) necessitates addressing this issue at a clinical level and intervening. It is important that the psychoeducation structured in this study significantly reduces aggressive tendencies among prisoners. Because ASPT have a taxonomy that includes aggression involving physical violence (i.e., fighting) and non‐aggressive behaviors (i.e., lying, cheating) (Bongers et al. 2004). It is clear that a significant proportion of prisoners in Türkiye have been convicted for violent offences involving physical assault (Türkiye İstatistik Kurumu 2020). Therefore, although there was no significant decrease in antisocial personality traits such as rule‐breaking, impulsivity, and lying, the finding of a significant decrease in aggression scores is very important. This decrease showed a significant effect in both the intra‐group (time‐dependent) and inter‐group contexts, demonstrating the effectiveness of the group‐based psychoeducation applied. Previous studies have reported that experimental interventions targeting ASPD have yielded varying results (Bateman et al. 2016; Hesse et al. 2022; Thylstrup et al. 2015; Wetterborg et al. 2020). The findings of this study revealed that a significant reduction was observed only in the aggression subdimension as a result of the intervention. This result may be directly related to the specific characteristics of the target group studied. This is because a significant proportion of the prisoners had been convicted of minor assault offences. This situation may also indicate that prisoners may be more motivated to control their aggressive impulses. Furthermore, the fact that the intervention programme's content included more intensive and powerful elements to facilitate a reduction in aggression may also have contributed to this result. Moreover, as aggressive behaviors are socially more visible and strongly rejected by society, it may have been easier for individuals to develop control over these behaviors compared to other antisocial tendencies. Furthermore, while an 8‐week intervention may lead to a reasonable reduction in aggressive tendencies that result in convictions, the more deeply ingrained traits of rule‐breaking, impulsivity, and lying may require longer‐term and more profound change processes. However, the study was conducted in prison conditions. In prison culture, negative processes that an individual experiences functionally in their life can become a defence mechanism. For example, lying can function as a type of adaptation strategy or coping strategy in the prison environment. Therefore, a short‐term experimental intervention may have been insufficient to reduce the complex and sophisticated functional structures underlying these negative behavioral tendencies.

The second finding of this study is that the decrease in aggression scores varies depending on levels of rule‐breaking. In other words, it was found that the significant decrease in aggression scores achieved through psychological interventions was moderated by prisoners' perceptions of rule‐breaking. Previous studies have indicated that ASPT, such as aggression and rule‐breaking, can be considered risk factors for each other (Jessor and Jessor 1977). Therefore, it seems reasonable that rule‐breaking is effective in psychological interventions targeting aggression. It was found that the dramatic decrease in aggression scores after the experimental intervention was much more pronounced in prisoners with low tendencies toward rule‐breaking. In other words, psychological interventions targeting prisoners with moderate to high levels of rule‐breaking resulted in a more limited decrease in aggression scores. No studies were found regarding the moderating effect of rule‐breaking on changes in aggression scores. However, there are studies showing that aggression and rule‐breaking together bring about change (Cheng et al. 2024) or that while aggression decreases, there is an increase in rule‐breaking (Givens and Reid 2019). This study indicates that individuals with low levels of rule‐breaking are able to control their aggressive tendencies thanks to the knowledge and skills they acquire through psychoeducation. Therefore, in any intervention targeting individuals with aggressive tendencies, priority should be given to clinical interventions with content that primarily reduces the tendency towards rule‐breaking. In other words, the effect of a psychological intervention programme that attempts to reduce aggressive tendencies may be limited in a target group consisting of individuals with moderate to high levels of rule‐breaking. This study also highlights the importance of examining how the relevant dynamic is shaped under specific conditions, rather than merely addressing a meaningful decline in a pathological dynamic in general clinical interventions. This perspective facilitates a more comprehensive and holistic approach to the findings obtained. Otherwise, the situation of spurious causality, which is a significant limitation of scientific studies, including experimental studies, may arise.

### 5.1 Limitations and Practical Implications

A key strength of this study is that it examined the effectiveness of group‐based psychoeducation with a more specific and specialized group, such as prisoners. However, this study has some limitations. First, due to prison conditions, the study was conducted with a limited number of prisoners. Studies conducted with a larger group of participants, which would increase the study's widespread impact and generalisability, would provide more comprehensive results. Although the random assignment of participants to the experimental and control groups is a strength of the study, it is known that individuals suffering from the aggression sub‐dimension of ASPT are more likely to end up in prison. In this context, it may be advisable to conduct studies with a group in which the other sub‐dimensions of ASPT are also found at medium and high levels in clinical measurements. Secondly, two measurements (pre‐test and post‐test) were taken in this study of prisoners. The use of follow‐up measurements in the experimental and control groups may raise doubts as to whether the findings represent a lasting effect. Thirdly, this study involved an intervention targeting ASPT. A psychological intervention targeting ASPD will yield strong clinical implications. Finally, to more effectively test the outcomes of experimental interventions, longitudinal qualitative data can be used in addition to quantitative measurements. Depending on the intervention, codes and themes derived from a semi‐structured interview form will provide much more detailed and in‐depth results. Furthermore, basing the effectiveness of the intervention on a single measurement is a limitation of this study. The reliability of the intervention outcomes can be increased by testing them against multiple sources.

This study has some clinical implications. Firstly, psychological interventions targeting ASPT, which are a significant predictor of criminal offences, have a constructive effect. Short‐term psychoeducation programmes can be developed for prisoners and individuals with antisocial tendencies at a clinical level. Furthermore, another finding in the study emphasizes that individuals' perceptions of rule‐breaking should not be overlooked in experimental interventions targeting aggression. This result indicates that it is vital to consider the interaction between the psychological dynamics to be addressed before commencing therapy. Furthermore, studies conducted on individuals with a strong tendency towards rule‐breaking may lead to the misconception that there is a dramatic decrease in aggressive tendencies. It will be necessary to examine the interactive effect of rule‐breaking, lying, and impulsivity with pre‐ and post‐test scores. This will provide a more comprehensive framework. The findings of the study showed no significant decrease in impulsivity, rule‐breaking, and lying, apart from aggression. This result may be a special circumstance arising from prison conditions. Indeed, as lying and rule‐breaking become survival strategies during the prison process, short‐term interventions may not produce effective results. Based on this finding, value‐based clinical interventions in prisons can be prioritized to enable longer‐term and more effective planning.

## 6 Conclusions

The 8‐week psychoeducation programme has the potential to reduce many ASPT in prisoners. However, the significant effect has been observed in the aggression sub‐dimension, which is a source of crime in judicial cases, and this has been shaped according to the individual's perception of rule‐breaking. It has been concluded that a decrease in aggression becomes meaningless in prisoners with medium to high levels of rule‐breaking, whereas this decrease is meaningful in prisoners with low levels of rule‐breaking.

## Author Contributions

**Mustafa Özmen**: conceptualization, investigation, writing – original draft, methodology, supervision, writing – review and editing, project administration. **Müjgan Kalabalık**: intervention, writing – review and editing.

## Funding

The authors have nothing to report.

## Data Availability

The data that support the findings of this study are available from the corresponding author upon reasonable request.
